# Automated Pipeline for Purification, Biophysical and X-Ray Analysis of Biomacromolecular Solutions

**DOI:** 10.1038/srep10734

**Published:** 2015-06-01

**Authors:** Melissa A. Graewert, Daniel Franke, Cy M. Jeffries, Clement E. Blanchet, Darja Ruskule, Katja Kuhle, Antje Flieger, Bernd Schäfer, Bernd Tartsch, Rob Meijers, Dmitri I. Svergun

**Affiliations:** 1 European Molecular Biology Laboratory (EMBL) Hamburg, 22607 Hamburg, Germany; 2 Robert Koch-Institut, Division of Enteropathogenic Bacteria and Legionella (FG11), Burgstr. 37, 38855 Wernigerode, Germany; 3 Malvern Instruments GmbH, Rigipsstr. 19, 71083 Herrenberg, Germany

## Abstract

Small angle X-ray scattering (SAXS), an increasingly popular method for structural analysis of biological macromolecules in solution, is often hampered by inherent sample polydispersity. We developed an all-in-one system combining in-line sample component separation with parallel biophysical and SAXS characterization of the separated components. The system coupled to an automated data analysis pipeline provides a novel tool to study difficult samples at the P12 synchrotron beamline (PETRA-3, EMBL/DESY, Hamburg).

Biological SAXS has become a streamlined technique to rapidly characterize overall structural parameters and conformational changes of proteins, nucleic acids and macromolecular complexes in solution^1^,^2^,^3^,^4^. However, for solutes containing aggregates, contaminating particles, partially dissociating complexes or oligomers, structural interpretation becomes difficult as most analysis methods for 3D structural analysis require monodisperse solutions^5^. Complementary techniques including size exclusion chromatography (SEC), analytical ultracentrifugation, native gel electrophoresis as well as dynamic and static light scattering are employed to ensure sample quality in advance for SAXS measurements. Indeed, the integration of an in-line SEC separation step (SEC-SAXS)^6^, where individual components are exposed to the X-ray beam while eluting from the column, provides evidence for data being collected from pure samples. Many state-of-the-art synchrotron beamlines now offer SEC-SAXS set-ups^7^,^8^,^9^,^10^ and SEC-SAXS has been conducted on intense laboratory sources^11^. An important application of SEC-SAXS is the analysis of the structure of solubilized membrane proteins which benefits from the removal of free micelles directly before the measurement^12^.

Standard batch SAXS measurements are now highly automated with robotic sample changers and automatic processing and analysis pipelines allowing for high throughput studies^13^. In contrast, SEC-SAXS experiments and their processing are still largely done interactively. The analysis of SEC-SAXS data is a major challenge given the large number (typically thousands) of scattering curves measured at continuously varying sample concentrations. Knowledge of the solute concentration is a must for the proper analysis of experimental SAXS data that involves subtracting background scattering contributions and normalizing the resulting intensities against the sample concentration. In addition, knowledge of the solute concentration also permits the determination of the solute species mean molecular weight (MW). The set-up presented here combines ultraviolet-visible light (UV-vis) and refractive index detectors to quantify the solute concentration of the flowing sample. In addition, the right angle light scattering (RALS) detector provides an independent means to estimate the MW of the eluted samples. The MW estimates integrated over a peak volume allow one to independently assess the oligomeric state of the eluting fraction, significantly improving the fidelity of subsequent interpretation steps. The combination of the biophysical and SAXS information allows for a full automation of SEC-SAXS processing and analysis.

## Results

**Figure 1:**
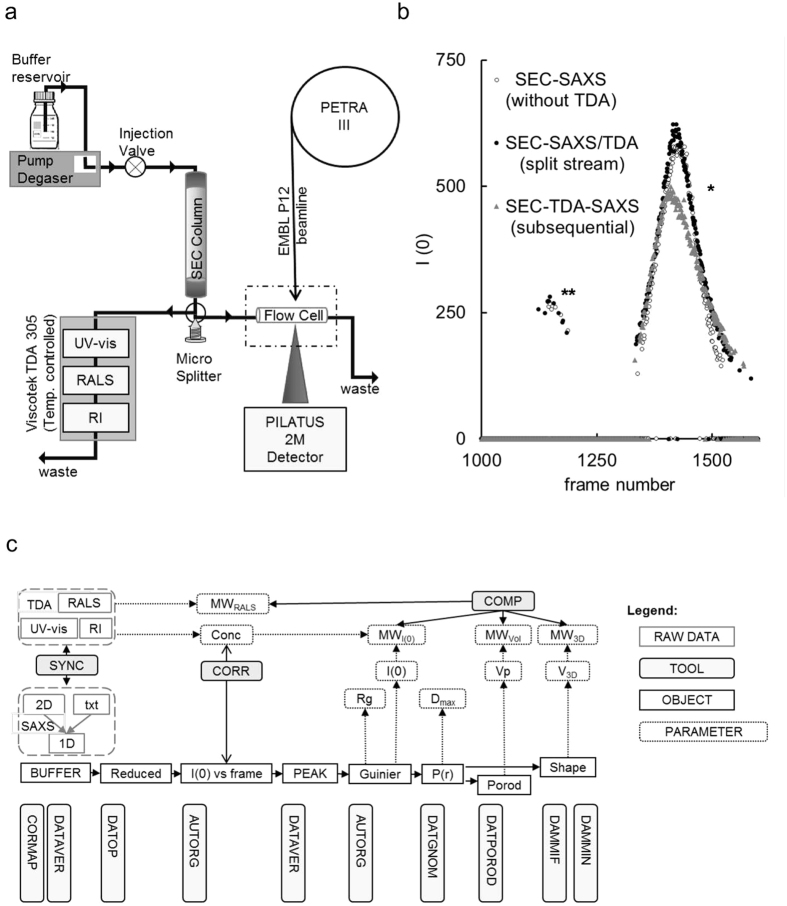
SEC-SAXS/TDA set-up at the EMBL-P12 beamline. (**a**) Using a micro-splitter valve, the eluting stream is divided in equal parts enabling in-parallel SAXS and light-scattering measurements.(**b**) Automated SEC-SAXS pipeline trace of *I*(0) vs. frame number. Bovine serum albumin was analyzed without TDA detectors (SEC-SAXS; ◯), with a parallel SEC-SAXS/TDA split stream set-up (●) and in-line SEC-TDA-SAXS set-up (△). *, ** indicate the peaks corresponding to the monomeric and dimeric fraction, respectively. (**c**) Schematic of the P12 data processing pipeline with integrated SEC-SAXS/TDA modules.

**Figure 2:**
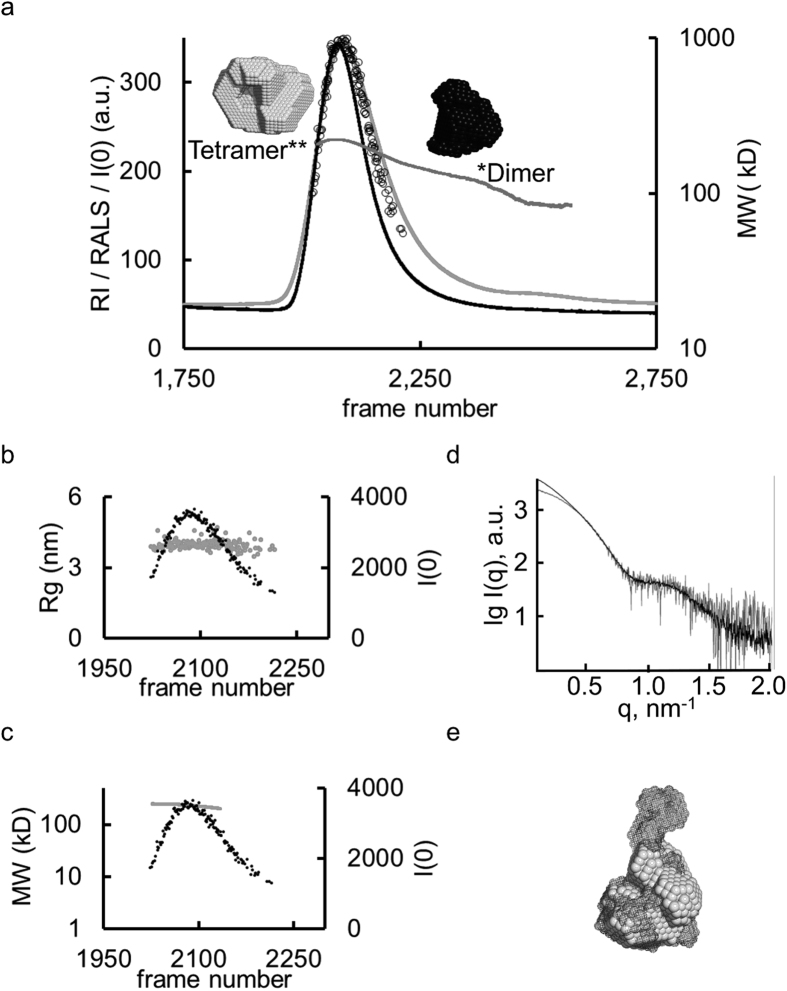
Biophysical and structural characterization of Lpn-PlaB. (**a**) The SEC-SAXS/TDA elution profile of Lpn-PlaB. RALS (black), RI (light gray) and the corresponding MW_RALS_ correlations (gray) across the elution peak. The oligomerization states are indicated and the 3D reconstructions presented. SAXS *I*(0) values determined from the SEC-SAXS pipeline are shown as open circles. (**b+c**) Determination of *R*_*g*_ and MW across the SAXS *I*(0) peak. The MW_RALS_ (230 ± 15 kDa) corresponds to the MW determined from the zero angle scattering by SAXS (225 ± 15 kDa). Due to weak scattering intensities only the tetrameric peak *I*(0) was detected automatically by the pipeline. (**d**) SAXS profiles of Lpn-PlaB collected in batch mode (black) and after component separation (tetramer (gray)) (**e**) the corresponding 3D reconstructions (inlay, as mesh and bead model, respectively).

The new SEC-SAXS setup at the EMBL P12 beamline (PETRA-3, DESY, Hamburg) utilizes a modular triple detector array (TDA, Viscotek model TDA 305, Malvern Instruments) that extracts MW estimates of SEC-separated components by correlating refractive index (RI) and/or UV-vis concentrations with RALS data (Fig. 1a). Initially, a simple in-series SEC-TDA-SAXS configuration was employed by attaching the TDA between the exit of the SEC column and the entry to the SAXS sample capillary. Despite small dead volumes of the three individual TDA chambers (10-12 μl), additional dilution of the eluting sample and, thus, peak broadening was observed (Fig. 1b, gray triangles). The decrease in sample concentration leads to a reduction of the absolute I(0) value (*) and can even hinder the detection of eluting components (e.g. the dimeric fraction of BSA; **). Further, the samples flowing through the RI detector experiences temperatures held at a default 5 °C above ambient (25 °C) and runs the risk of being affected by increased temperature before the SAXS measurements. Therefore, the mobile phase was split immediately after the SEC column using a micro-splitter valve (P-451 Upchurch Scientific®, Fig. 1a) to divert the flow equally between the TDA and SAXS capillary (SEC-SAXS/TDA). This parallel configuration enables the determination of component concentrations and corresponding MW_RALS_ using the TDA with simultaneous SAXS data collection from the same components that are unaffected by dilution or heating. The post-SEC valve has a 2.8 μl dead volume and allows the accurate splitting of the low-flow stream despite the internal backpressure of the TDA detector (0.2 MPa). As the splitting only reduces the flow rate and not the sample concentration, there is no loss in absolute X-ray (Fig. 1b) or light scattering intensities (Supplement Fig. S1). However, even though the reduced flow rate has *per se* no negative effect on the X-ray scattering intensities, caution must be taken to assess the susceptibility of the sample to radiation damage due to the slower removal from the irradiated area. This can be tested on small aliquots of sample (10-20 μl) in ‘batch mode’ prior to SEC-SAXS/TDA (Fig. 2d) and, if necessary, compensated for via beam attenuation or addition of small molecules to the mobile phase such as 5% (v/v) glycerol.

In addition, for non-aggregated mixtures (e.g., oligomeric-equilibrium samples), batch-mode SAXS can be useful to obtain a base line dataset where it may be possible to deconvolute the mixture profile using the separated component SEC-SAXS data (see chapter 5 in Ref. 4) and thus help identify potential structural changes caused by component association/disassociation, or unfavorable interactions with the column matrix. In these difficult cases additional biophysical characterization is recommended (e.g., small-angle neutron scattering, dynamic light scattering, circular dichroism, nuclear magnetic resonance spectroscopy) to supplement the results and further the interpretation of combined batch-mode/SEC-SAXS experiments.

New software modules have been added to the beamline data analysis and modelling pipeline^13^ to automate user operation (Fig 1c). Briefly, after synchronized data collection, frames containing solely the scattering of buffer are identified, averaged and then subtracted from each acquired data frame to produce net scattering from the solute. Using Auto*R*_*g*_^14^, the forward scattering *I*(0) (proportional to MW) and radius of gyration (*R*_*g*_) are automatically extracted for each subtracted frame and correlated with the TDA output (the latter provides RI-based concentration and MW_RALS_ estimates). Each reduced SAXS profile is normalized to concentration and frames with consistent *R*_*g*_ values are averaged to produce final SAXS scattering curves corresponding to the separated sample components. These data are passed to down-stream modules for further determination of overall parameters and *ab initio* shape modelling. Finally, MW estimates from different approaches (SAXS and RALS) are compared for consistency.

**Figure 3:**
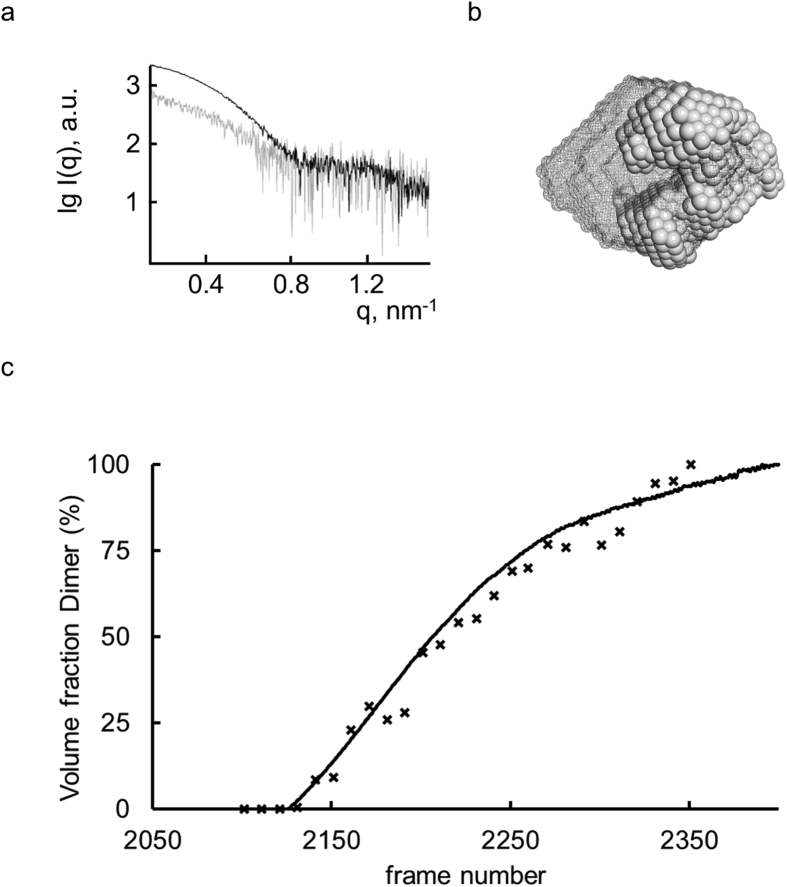
Analysis of dimeric PlaB with SAXS and TDA data. (**a**) SAXS scattering profiles of Lpn-PlaB collected from the tetrameric fraction (black, frames 2000-2160) and dimeric fraction (gray, frames 2350-2360). (**b)** 3D reconstructions of the tetrameric fraction (dark mesh) and bead model of the dimer fraction (gray). For these, the SAXS profiles shown in (a) were merged with data collected at higher concentration in batch mode (4.5 mg/ml, Fig. 2d) and 10 runs of DAMMIF were performed to generate a starting model for a final run of DAMMIN. (**c**) Estimation of the volume fraction of PlaB dimeric species. The volume fraction of the dimeric component in the respective SAXS frames was determined with Oligomer (crosses). For this 10 frames were averaged and fitted by distinct ratios of the form factors derived from the scattering profiles corresponding to the tetrameric and dimeric species as shown in (**a**). This coincides very well with the volume fractions derived from the TDA data (black curve); the % volume fraction of dimeric species was determined by the ratio (MW_tetramer_ – MW_RALS_) to MW_dimer_.

We tested the automated SEC-SAXS/TDA set-up on a number of commercially available proteins with varying polydispersity. The accuracy of MW_RALS_ estimations of the major components of each sample is within the range of error (5-10% as stated by the manufacturer, Supplementary Table 1). The quality of the SAXS data is demonstrated by the excellent agreement between the structural parameters expected for monodisperse particles and the spatial alignments of SAXS-based *ab initio* models and the respective crystal structures (Supplementary Fig. 2+3). User projects have also profited from the new set-up (Fig. 2). The MW of phospholipase PlaB of *Legionella pneumophila* (Lpn PlaB) determined from the TDA and SAXS data (MW_RALS_= 230 ± 15 kDa; MW_I(0)_ = 225 ± 15 kDa) across the main SEC elution peak corresponds to the MW expected for a tetramer (220 kDa, Fig. 2a+c). Surprisingly, in addition to the major tetrameric fraction a small plateau in the RALS derived MW trace at 120 ± 10 kDa suggests that a dimeric component is also present at low concentrations. SAXS frames corresponding to the TDA elution peak (dimer plus tetramer) were processed by the automated pipeline (Fig. 3) and the mole-fractions of the dimer:tetramer equilibrium were determined using Oligomer^15^. There is an excellent agreement with those mole-fractions independently calculated from the TDA RALS and RI traces, demonstrating that the SAXS and TDA measurements are acquired in parallel from identical separated sample components. The observed equilibrium has significant biological implications as it has been shown that the virulence factor Lpn-PlaB assembles as inactive tetramers at micromolar concentrations and undergoes activation upon disassociation at low concentration^16^. The data presented here suggest that this activation–deactivation mechanism could occur via a dimeric intermediate. Given the low concentration and the transient nature of the dimer, it is highly unlikely that this species would have been structurally characterized using standard methods.

## Conclusion

In conclusion, we have developed a SEC-SAXS/TDA system for parallel SAXS and biophysical measurements to validate and correlate the molecular weights of separated sample components for automated data analysis and modelling of polydisperse macromolecular samples. The system separates non-specific aggregates and other contaminating species from a target of interest while also offering a tool to investigate systems driven by concentration-dependent equilibrium. Thus, this set-up allows the analysis of complicated systems such as transiently formed complexes, weakly associated complexes as well as composite samples for example protein:nucleic acids complexes and proteins with detergents or lipids. The analysis of the latter is of growing interest regarding the structure of solubilized membrane proteins. The analysis of proteins which interact with co-solutes such as glycerol, sucrose, lipids and salts is of special interest for the pharmaceutical industry in respect to formulation studies. However, a critical point in the assessment of the concentration and molecular weight of all these difficult cases is the accurate determination of the of the refractive index increment dn/dc. Not only is the RI signal proportional to this value, but it is also an important term in light scattering calculations as the intensity of scattered light is directly proportional to its square. Most importantly, this value changes with the molecular composition of the sample and is, thus, altered in the cases mentioned above, and can be calculated by using all three individual detectors incorporated in the TDA. Whereas the RI detector has the advantage for the determination of concentrations in an automated manner (as this signal is not sequence dependent), the UV-vis detector can be employed to assess either the stoichiometry of multicomponent complexes or their solute concentration using pre-defined wavelength-dependent extinction coefficients. With the knowledge of the solute concentration on the other hand, the altered dn/dc increment can be determined from the RI signal and hence all the parameters are available to determine molecular weight from the light scattering signal. We performed correlation measurements using RI and OD experiments on the same stock solutions using table-top instruments to derive the correlation constant for proteins (Supplementary Fig. 4). Once more data has been collected on such difficult systems with the SEC-SAXS/TDA set-up, it will be interesting to see if an automatic assessment of an altered dn/dc increment could be useful for downstream calculations as well as identifying protein samples with additional bound ligands. For composite samples, the SAXS- and light scattering/absorption/refraction-determined MWs may disagree with each other, and therefore observing inconsistencies between these values may point to the composition of the sample under study. Another advantage of the use of the described set-up in formulation studies, is the possibility to assess correct scattering frames for background subtraction by monitoring baseline fluctuations of the RI signal, which is a very sensitive tool for altering solvent compositions. Especially for the SAXS analysis with frequently added co-solutes such as glycerol, sucrose and lipids, correct background subtraction is of immense importance as these solutes also affect the contrast of the SAXS signal.

In this respect, the possibility arises, if the fluctuating RI signal could also assist in determining suitable regions for SAXS background subtractions when using alternative purification strategies. Whereas the advantages of using for example ion-exchange, reverse phase or hydrophobic interaction chromatography in an online SAXS mode are apparent in the potential for analyzing a number of so far inaccessible samples, however, the major challenge of such an experiment obviously concerns the correct background subtraction. Future studies will be necessary using the parallel TDA/SAXS setup described here to evaluate the correlation between RI-SAXS data measured from both blank and sample gradient-SAXS experiments to extend the application of the split-stream TDA/SAXS method beyond standard size exclusion.

## Methods

### Sample preparation

Human serum albumin (HSA), bovine serum albumin (BSA), sweet potato ß-amylase, bovine erythrocyte ubiquitin and horse spleen apoferritin were purchased from Sigma-Aldrich. Bovine pancreatic ribonuclease A, chicken egg white conalbumin (ovotransferrin), bovine liver catalase and chicken ovalbumin were acquired from GE Healthcare. For SEC-SAXS/TDA analysis the samples were dissolved in the respective SEC buffers at the desired concentrations (approximately 5 mg/ml). If not stated otherwise, the SEC buffer contained: 100 mM Tris, pH 7.5, 150 mM NaCl, and 5% v/v glycerol. All samples were filtered through 0.2 μm centrifugal filter units (Millipore) prior to loading on to the respective SEC column (see below). SEC experiments were performed at a flow rate of 0.4 ml/min and at room temperature. A Superdex 200 column (10/300, GE Healthcare) was used for all data presented here. Final solute concentrations were determined by measuring the absorbance at 280 nm (Thermo Scientific NanoDrop ND-1000) and using calculated extinction coefficients expressed as ε1% (10 mg/ml) from ProtParam^17^: ε_HSA _= 5.8, ε_BSA_ = 6.14, ε_ß-amylase_ = 17.65, ε_ubiquitin_ = 1.74, ε_ribonuclease_ = 6.53, ε_conalbumin_ = 11.38, ε_ovalbumin_ = 7.32. The concentration of catalase was estimated by carefully determining the dry weight of protein powder added to solution while the concentration of apoferritin stock solution was given by the manufacture. Recombinant phospholipase A PlaB of *Legionella pneumophila* was prepared as described in Kuhle *et al.*, 2014^16^) using a Strep-Tactin Superflow high capacity matrix (IBA).

### TDA runs

The separated sample components from in-line size exclusion chromatography (SEC) were analysed using combined right-angle light scattering (RALS), refractive index (RI) and UV-vis measurements (UV) made with a triple detector array (TDA, Viscotek model TDA 305 (Malvern Instruments Ltd., Malvern, UK)). The TDA data were processed using the integrated Omnisec software. The molecular weight (MW_RALS_) of each species eluting from the SEC column was assessed using correlated concentration (*c*) measurements derived from base-line corrected RI in combination with base-line corrected RALS intensities calibrated against a bovine serum albumin narrow (monomeric) standard (RALS = *c.*(dn/dc)^2^.*MW*.k_RALS_ and RI = *c.*(dn/dc).k_RI_ where dn/dc is the refractive index increment of unmodified protein, 0.185 mL.g^−1^ and k_RI_ and k_RALS_ are the TDA instrument calibration constants). By default, we employ RI for estimating protein concentration as it has advantages over UV absorption methods^18^ and can be used as a very sensitive tool to monitor baseline fluctuations for the selection of a suitable region for SAXS background subtractions.

It should be noted, that detector calibration parameters had to be redesigned for the split stream set-up, as these are derived through integration of the elution peak of the standard protein, and thus alter with the reduction in total protein amount. MW_SEC_ was determined with the conventional calibration method (also integrated in the Omnisec software) for which a calibration curve based on the retention time of proteins with known MW is generated for each individual column.

### SAXS data collection and processing

SAXS data were acquired using an incident beam size of 200 × 110 μm^2^ (FWHM) in a 1.7 mm quartz capillary held under vacuum. The SAXS intensities were reduced to I(q) vs q, where q = 4πsinθ/λ where 2θ is the scattering angle and the wavelength λ = 0.124 nm (10 keV) using the integrated analysis pipeline SASFLOW^13^. The q-axis was calibrated with silver behenate and the resulting profiles were normalized for exposure time and sample transmission. For samples with increased risk of radiation damage, the incident beam intensity was attenuated by a factor of seven (from 5.1 × 10^12^ to 7.3 × 10^11^ photons.s^−1^) by moving a 300 μm aluminium foil into the incident beam path. Two different modes were employed to collect SAXS data. In a standard ‘batch´ mode, the samples were loaded by an automated sample changer. Twenty successive exposures (50 ms each) were collected and processed by the integrated data analysis pipeline. Data collected on BSA were subsequently used to calibrate the forward scattering intensities at zero angle, I(0), for other batch measurements as well as for the data obtained from the SEC-SAXS/TDA experiments. For the SEC-SAXS and SEC-SAXS/TDA measurements, either the full or partial eluent low-flow stream coming from the SEC column was diverted through the capillary while 1000–4000 individual frames were collected with 1 s exposure.

Although the reduced flow-rate of the split stream through the capillary has no negative effect on the X-ray scattering intensities (Fig. 1b), caution must be taken to assess the susceptibility of the sample to radiation damage due to the slower removal from the irradiated area. This can be tested on small aliquots of sample (10–20 μl) in ‘batch mode’ prior to SEC-SAXS/TDA and, if necessary, compensated for via beam attenuation or addition of small molecules to the mobile phase such as 5% (v/v) glycerol^19^,^20^.

Besides the automated processing pipeline (see supplementary information), additional programs of the ATSAS package^21^ were used such as Oligomer^15^ to assess the volume fractions of dimeric PlaB during the SEC-SAXS/TDA run and SUPCOMB^22^ to spatially align the available crystal structures with the generated *ab initio* models obtained from SEC-SAXS/TDA data. Model representations displayed in the figures were generated with Pymol Molecular Graphics System (Schrödinger, LLC.).

## Additional Information

Accession codes: The SAXS data and model of phospholipase PlaB of *Legionella pneumophila* (Lpn PlaB) in tetrameric state have been deposited at SASBDB (www.sasbdb.org), accession code: SASDA97.

**How to cite this article**: Graewert, M. A. *et al.* Automated pipeline for purification, biophysical and X-ray analysis of biomacromolecular solutions. *Sci. Rep.* **5**, 10734; doi: 10.1038/srep10734 (2015).

## Supplementary Material

Supplementary Information
