# Supplementary material for: Automated Pipeline for Purification, Biophysical and X-Ray Analysis of Biomacromolecular Solutions
Source: Sci Rep. 2015 Jun 1;5:10734. doi: 10.1038/srep10734 (PMC5377070; doi:10.1038/srep10734)
Supplement: Supplementary Information [file srep10734-s1.pdf]

Automated pipeline for purification, biophysical and X-ray analysis of biomacromolecular solutions

Melissa A Graewert[1*], Daniel Franke[1], Cy M. Jeffries[1], Clement Blanchet[1], Darja Ruskule[1], Katja Kuhle[2], Antje Flieger[2], Bernd Schäfer[3], Bernd Tartsch[3], Rob Meijers[1]and Dmitri I. Svergun[1*]

[1]European Molecular Biology Laboratory (EMBL) Hamburg, 22603 Hamburg, Germany

[2]Robert Koch-Institut, Division of Enteropathogenic Bacteria and Legionella (FG11), Burgstr. 37, 38855 Wernigerode, Germany

[3]Malvern Instruments GmbH, Rigipsstr. 19, 71083 Herrenberg, Germany

*Corresponding Authors

Melissa Graewert
EMBL Hamburg
Notkestrasse 85, Geb. 25 A
22603 Hamburg, Germany
Tel: +49 [0] 40 89902 115
graewert@embl-hamburg.de

Dmitri Svergun

EMBL Hamburg
Notkestrasse 85, Geb. 25 A
22603 Hamburg, Germany
Tel: +49 [0] 40 89902 125
svergun@embl-hamburg.de

Supporting information

S1. Automated SAXS data collection and analysis.

For automatic data collection and analysis, the pipeline currently in operation at P12[1] was extended with additional modules specifically for SEC-SAXS/TDA experiments (Fig. 1c). User intervention for the individual runs is, thereby, kept to a minimum. The user simply loads the sample to the chromatography system (recording the volume), abides to the standard safety measurements, and sets the interlock system of the experimental hutch before starting the data collection (SYNC). The required input parameters primarily consist of the name of the sample, the desired exposure time (default 1 sec) and number of frames (typically between 1000–4000 frames), which depends on the chromatographic separation step, i.e. volume of the column and reduced delivery flow-rate (typically 0.2–0.3 ml/min). Submission of the run parameters to the beamline meta server (BMS) results in the instant collection of TDA data. The user can decide, if the SAXS data should be collected immediately or closer to the time point at which the protein of interest is expected to elute. For the latter, the possibility to tell the BMS to wait for the detection of a rise in RI signal before triggering data acquisition has been implemented.

During the SEC-SAXS/TDA run, the BMS checks a configurable file system location for incoming data files and commences the radial averaging of two-dimensional images (2D) to one-dimension curves (1D) with incorporation of the information for the header (txt). Once a run is completed and all the 1D files are generated, the data processing pipeline is started. The first step in data processing is the subtraction of the solvent blank to obtain the scattering from the macromolecules. For this, frames comprising only buffer components are identified (BUFFER). Frames collected at the beginning of the run are evaluated in terms of their probability of similarity by comparison of the respective correlation maps (CORMAP).[2] In some cases, frames closer to the elution peak are more suitable for buffer subtraction and can be determined by inspection of the RI signal, which is very sensitive for changes in buffer during the SEC run. Statistically similar data frames are averaged (DATAVER) and then subtracted (DATOP) from each acquired data frame to produce reduced scattering profiles (Reduced). Running AUTORG for each frame allows the determination of the forward scattering I(0) and R_g, which can be plotted against the respective frame number from the automatically generated I(0) vs frame csv file. This plot can be used to evaluate the successful outcome of the experiment (e.g., stability of R_g across an elution peak (Fig. 2b)). This plot is also used to correlate the SAXS data with the RI_{TDA} data (CORR). The TDA elution profile data is adjusted to the same volume scale as the number of SAXS data frames by shifting the trace along the x-axis to obtain an overlay of the I(0) with the RI elution profile which is directly proportional to the

concentration of the eluting components. At this point it should be noted that the TDA output file can be automatically generated with the Omnisec software (Malvern Instruments Ltd., Malvern, UK). It is, however, recommended that the user check and process the TDA data with the available Omnisec software, as the accuracy of the molecular weight estimation (MW_{RALS}) and concentration improves by manually setting baselines and integration limits. Correlation of the SAXS and TDA data allows RI concentration to be extracted for each frame, so that the respective scattering profile can then be normalized to concentration, from which $MW_{I(0)}$ can be assessed and validated against MW_{RALS} from the TDA. Frames corresponding to the component of the SEC elution peak and with consistent R_g values are averaged to produce the final reduced SAXS profile (PEAK). This final file is passed on for further processing to determine the overall structural parameters of the averaged scattering data obtained from the sample component such as R_g and I(0) from the Guinier fit (AUTORG)[3,4] the p(r) function and D_{max} using DATGNOM[3,5] as well as the excluded volume estimates from the Porod volume (Vp, DATPOROD) and 3D *ab initio* reconstructions from which MW_{Vp} and MW_{3D} can be estimated. Shape determination is performed through 10 runs of DAMMIF[6] from which a starting model is derived through DAMAVER[7]. A final run of DAMMIN[8] using this starting model leads to a well refined model that can be studied to gain information on the shape of the molecule. With the tool COMP, an output file is generated for rapid comparison of all the MW values and the possibility to assess the success of the experiment.

S2. Comparison of different methods for molecular weight estimations.

The emphasis of this research is to provide a method to separate components of polydisperse systems into their respective monodisperse components so as to increase the confidence in MW determinations and the subsequent analysis of measured SAXS data. Assessing MW is a key step for the interpretation of biological solution SAXS data.[9] The MW estimates of the components of a sample can be derived and cross-correlated with the estimates from SAXS by collecting additional biophysical data of the analysed components using the TDA. It is important to understand on what assumptions these MW estimates are made and define their accuracy.

S2.1. SAXS-based MW estimates.

The molecular weight (MW) of a component can be estimated from the SAXS data via:

S2.1.1. The forward scattering intensities at zero angle, I(0).

The scattering intensity at zero angle I(0) obtained from the Guinier approximation[3,4] or from the real-space distance distribution, p(r)[10], is directly associated with the volume and scattering length density of a particle and can thus be used to determine the molecular weight of monodisperse samples. The determination of the MW from I(0), $MW_{I(0)}$,can be performed by calibrating the sample scattering relative to a standard with the same scattering length density. For example, the I(0) determined from standard proteins with known molecular weights such as lysozyme[11], bovine serum albumin[12] or glucose isomerase[13] can be used for calibration and estimation of the molecular weights of protein samples. This estimation is, however, strongly dependent on accurate determination of the concentrations of the protein sample as well as the standard protein used for the calibration. The accuracy of the MW determination using this method has been estimated to be around 10-15%.[14] Similar, Lupolen[15] or water[16] can be used for an absolute calibration of the scattering intensities (I(q), cm^{-1}), however, here the estimation of the partial specific volume of the protein (that can be calculated from the primary amino acid sequence, e.g., using NucProt[17] may incur a source of error.

For the data collected with the SEC-SAXS/TDA set-up described here, I(0) values automatically determined from each processed frame were normalized based on a batch measurement of BSA at known concentration. $MW_{I(0)}$ was then determined by deriving I(0) from the final PEAK scattering profile combined with the corresponding concentration determined from RI measurements from the TDA.

S2.1.2. From excluded volume MW_{Vp} and MW_{3D}

The MW estimations based on Porod volume (Vp, MW_{Vp}) or from a derived ab initio bead model (MW_{3D}) are computed without the necessity to normalize the SAXS data against a known standard. Consequently these MW estimates are not dependent on accurate concentration estimates. However, a number of other factors such as particle anisometry and flexibility influence the relationship between MW and the excluded volume. [3,18] Nevertheless, with an accuracy of around 20%, MW_{Vp} can be automatically calculated with the program DATPOROD[3] which determines the exclude volume based on Porod's law.[10] In a similar manner the MW can be estimated from the excluded volume of *ab initio* 3D reconstructions (MW_{3D}). For this the volume of the beads as well as the interfering gaps are summed up and are reported in the output files of programs such as DAMMIF and DAMMIN. For proteins, dividing the obtained volume (V_{3D}) of the bead model by 2 provides the estimate of the MW of the scattering particle, again with an accuracy of approximately 20%.[3]

S2.2. MW estimates from standardized SEC and with the described TDA set up.

The molecular weights estimated from the SAXS data can be validated against the MW estimates from SEC, either using a standardized SEC-column or from RI(UV)/RALS data obtained from the TDA.

S2.2.1. Standardized SEC column MW estimates (MW_{SEC}).

The separation of components with size exclusion chromatography relies on different migration behaviour of molecules through a media of porous beads.[19] Thus, small and globular proteins penetrate these pores more easily, and elute from the column with an increased retention time compared to larger molecules. Any SEC column can be calibrated with 4–5 standard proteins and comparison of the elution volumes can provide a rough estimation of the MW_{SEC}, without the need for RALS measurements. However, separation relies on the hydrodynamic volume and not MW and the migration behaviour can be altered through interactions of the column matrix with the mobile phase. Consequently, the MW estimation from a calibrated SEC column, based only on retention time, can be erroneous. For example, PlaB, analysed in this study, displays an increased retention volume (main peak at 10.8 ml). The elution volumes of the different oligomeric components of BSA with this set-up are: monomer (66 kD) at 12 ml, dimer (132 kD) at 10.5 ml, and trimer (197kD) at 9.45 ml. Thus, according to the PlaB elution profile, one would draw the conclusion that it is a dimer (Fig. 2a). However, when the MW is determined from both SAXS data and from RI/RALS measurements from the TDA, PlaB is unambiguously determined as a tetramer.

S2.2.2. MW estimates from the TDA data (MW_{RALS})

The inclusion of the TDA for molecular weight validation of the components of a sample eluting from a SEC column overcomes the issues of relying on column retention times to estimate the molecular mass of a sample. For particles in the size range of proteins, Rayleigh light scattering principles essentially apply so that scattering intensities recorded at 90° (RALS) relate to the size of a macromolecule in solution. Similar to SAXS, these RALS intensities have to be normalized to particle concentration and calibrated against a MW standard (e.g., BSA) with a known concentration and injection volume to obtain a MW_{RALS} estimate. With the correlation between the RALS signal and RI (or UV) concentration MW_{RALS} can be estimated across an elution peak independently from the retention volume and the stability of this MW estimate can be assessed across the peak. The error in the MW determination depends on the stability of the RI(UV)/RALS correlation and the chosen range used to perform the calculations. The range of error is between 5–10% in our experience.

Summary of various methods for MW estimation

	Basic principle	Major source of error
$MW_{I(0)}$	Forward scattering at zero angle is proportional to the volume squared and concentration of the solute and thus relates to the molecular weight of the solute.	Requires accurate estimation of solute concentration and knowledge of the partial specific volume. Contaminations, especially large species, contribute to the scattering.
MW_{Vp}	For globular proteins MW is proportional to the excluded volume of hydrated particles, which can be determined from the Porod invariant.[3]	Ratio between volume and MW is not always consistent, especially for anisotropic or unfolded/flexible particles. Quality of MW estimates are dependent on the angular range of the collected data.
MW_{3D}	For globular proteins MW is proportional to the volume of hydrated particles, which can be determined from 3D reconstructions.	Ratio between volume and MW is not always consistent. The $V_{3D}/2 = MW_{3D}$ relation becomes inaccurate if a particle is comprised of a scattering length density different to a protein or a mixture of scattering length densities (e.g., protein/DNA complex).
MW_{SEC}	Comparison of migration through a mobile phase consisting of porous particles.	Separation based on hydrodynamic volume. Influenced by attractive or repulsive interactions between the column matrix and mobile phase.
MW_{RALS}	Ratio of RI to RALS signal is directly proportional to the averaged molar mass	Contaminating species, if they are not well resolved by the SEC column, contribute to the scattering signal.

References

1. Franke, D., Kikhney, A. G. & Svergun, D. I. Automated acquisition and analysis of small angle X-ray scattering data. *Nucl. Instrum. Meth. A* **689**, 52-59 (2012).
2. Franke, D., Jeffries, C.M. & Svergun, D.I. (2015). *Nat Meth*. *Submitted*.
3. Petoukhov, M. V., Konarev, P. V., Kikhney, A. G. & Svergun, D. I. ATSAS 2.1 - towards automated and web-supported small-angle scattering data analysis. *J. Appl. Cryst.* **40**, s223-s228 (2007).
4. Guinier, A. La diffraction des rayons X aux tres petits angles; application a l'etude de phenomenes ultramicroscopiques. *Ann. Phys. (Paris)* **12**, 161-237 (1939).
5. Svergun, D. I. Determination of the regularization parameter in indirect-transform methods using perceptual criteria. *J. Appl. Cryst.* **25**, 495-503 (1992).
6. Franke, D. & Svergun, D. I. DAMMIF, a program for rapid ab-initio shape determination in small-angle scattering. *J. Appl. Cryst.* **42**, 342-346 (2009).
7. Volkov, V. V. & Svergun, D. I. Uniqueness of ab initio shape determination in small angle scattering. *J. Appl. Cryst.* **36**, 860-864 (2003).
8. Svergun, D. I. Restoring low resolution structure of biological macromolecules from solution scattering using simulated annealing. *Biophys. J.* **76**, 2879-2886 (1999).
9. Jacques, D. A. & Trewhella, J. Small-angle scattering for structural biology—expanding the frontier while avoiding the pitfalls. *Prot. Sci.* **19**, 642-657 (2010).
10. Porod, G. in Small-angle X-ray scattering (eds . *Glatter, O. & Kratky, O.)* 17-51. (*Academic Press,* 1982).
11. Hammel, M. *et al.* Solution structure of human and bovine beta(2)-Glycoprotein I revealed by small-angle x-ray scattering. *J. Mol. Biol.* **321**, 85-97 (2002).
12. Petoukhov, M. V. & Svergun, D. I. New methods for domain structure determination of proteins from solution scattering data. *J. Appl. Cryst.* **36**, 540-544 (2003).
13. Kozak, M. Glucose isomerase from Streptomyces rubiginosus - potential molecular weight standard for small-angle X-ray scattering. *J. Appl. Cryst.* **38**, 555-558 (2005).
14. Mylonas, E. & Svergun, D. I. Accuracy of molecular mass determination of proteins in solution by small-angle x-ray scattering. *J. Appl. Cryst.* **40**, s245-s249 (2007).
15. Kratky, O., Pilz, I. & Schmitz, P. J. Absolute intensity measurement of small angle x-ray scattering by means of a standard sample. *J.Colloid Interf. Sci.* **21**, 24-34 (1966).
16. Orthaber, D., Bergmann, A. & Glatter, O. SAXS experiments on absolute scale with Kratky systems using water as asecondary standard. *J. Appl. Cryst.* **33**, 218-225 (2000).
17. Voss, N. R. & Gerstein, M. Calculation of standard atomic volumes for RNA and comparison with proteins: RNA is packed more tightly. *J. Mol. Biol.* **346**, 477-492 (2005).
18. Watson, M. C. & Curtis, J. E. Rapid and accurate calculation of small-angle scattering profiles using the golden ratio. *J. Appl. Cryst.* **46**, 1171-1177 (2013).
19. Ricker, R.D. & Sandoval, L.A. Fast, reproducible size-exclusion chromatography of biological macromolecules *J. Chromatogr. A* **743,** 43-50 (1996).

Supplement Figure 1

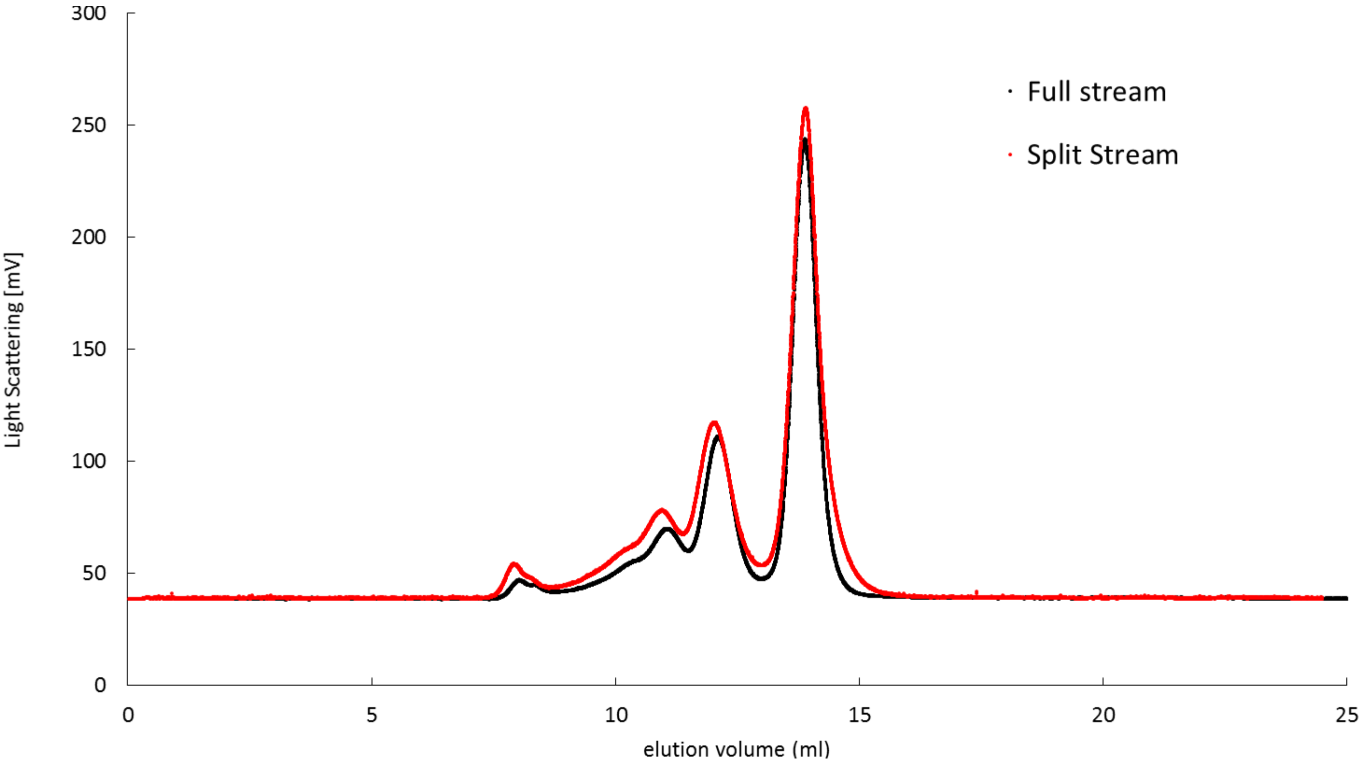

Figure S1 Comparison of the light scattering signal with full and split stream. 100 µl BSA (4.5 mg/ml) were passed over a Superdex 200 10/300 column equilibrated in 100 mM Tris, pH 7.8, 150 mM NaCl, and 5 % glycerol. Either the full eluting stream was passed directly to the TDA detectors (black curve) or partial stream by splitting the eluent with a micro-valve (red curve). The profiles coincide nicely demonstrating that neither the resolution of the separation nor absolute signal are effected by the splitting of the stream.

Supplement table 1: MW estimations (in kD)

For better comparison, data is shown as bar diagram below (see online methods for details on error estimation)

	MW_{Exp}	MW_{RALS}	MW_{SEC}	$MW_{I(0)}$	MW_{Vp}	MW_{3D}
Estimated error	±0 %	±7.5 %	±10 %	±10 %	±20 %	±20 %
Ubiquitin	8.5	8.5	11.9	8.3	6.1	7.35
Ribonuclease A	13.7	13	10	20.4	9.4	11.2
Ovalbumin	45	48.2	41.4	43.8	47.4	46.6
Human SA	66.5	65.4	81	68	63.2	70
Conalbumin	75	78.9	65	70	65.1	75.2
Beta Amylase	200	248	255	257.3	179	190
Catalase	232	258	210.6	257.3	176	188
Apoferritin	440	494	n.d	603.3	414	421.15

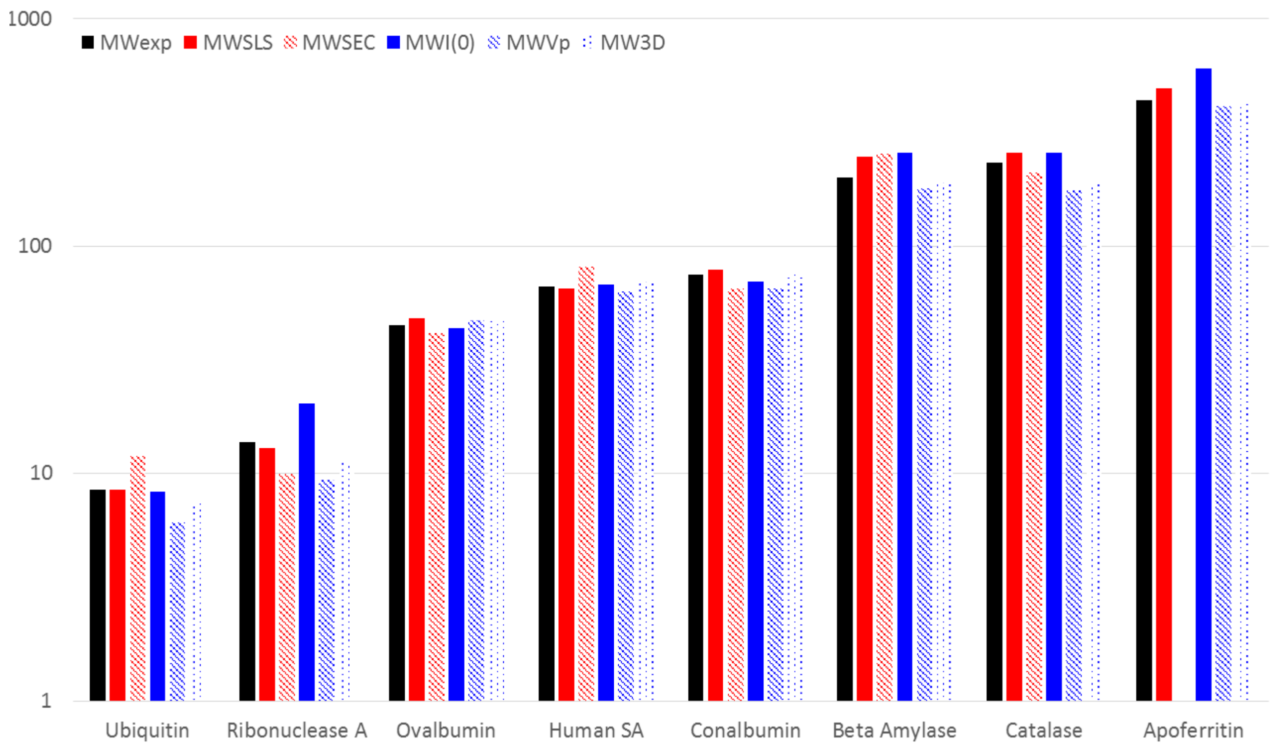

Supplement Figure 2

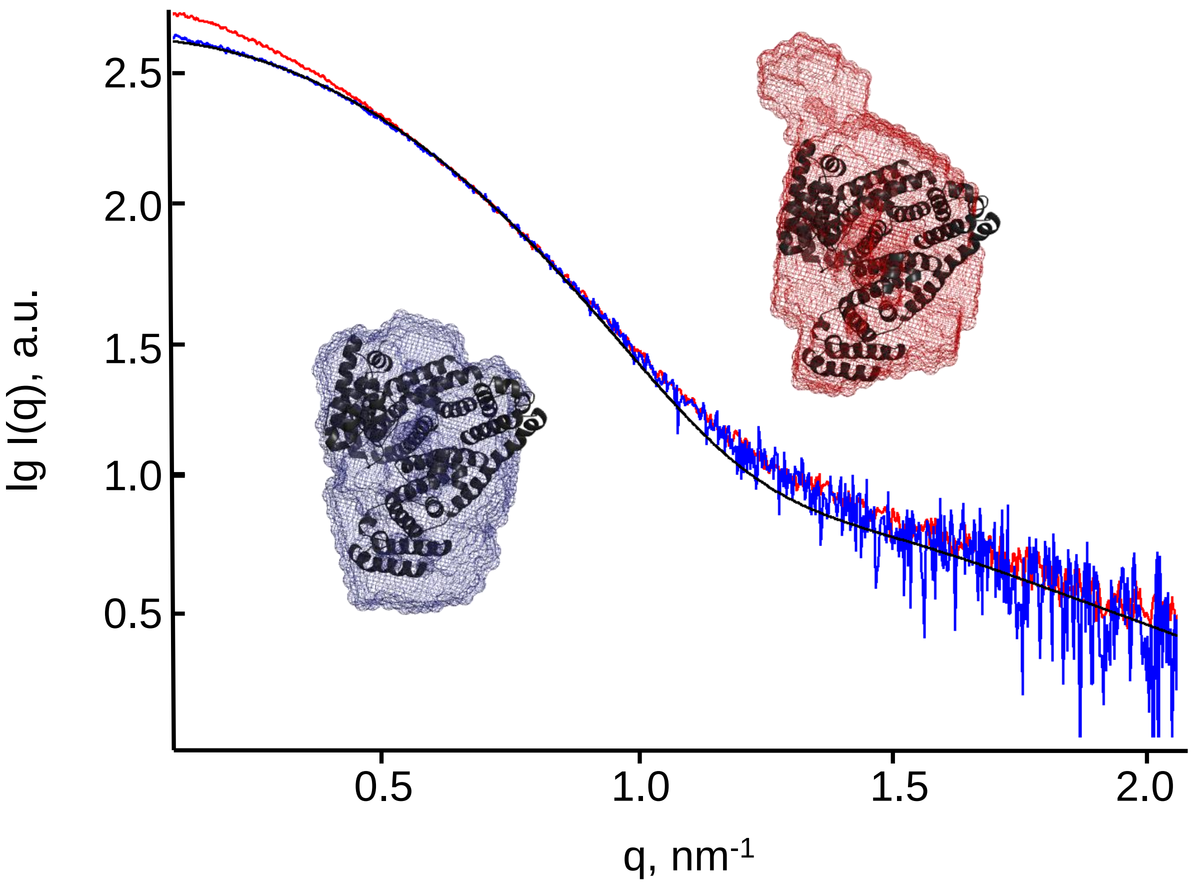

Figure S2 Improved quality of the SAXS data after component separation. SAXS profiles of conventional batch measurement of BSA (red, 4.5 mg/ml) and the averaged SAXS data frames corresponding to the SEC monomeric elution peak (blue) are compared with the theoretical SAXS scattering curve of BSA (black, pdb:code 3V03). The inlays show the 3D reconstructions (10 rounds of Dammif and a final round of Dammin) overlaid with the crystal structure. Note, that *the ab initio* model of the batch measurement shows a typical extension that arises from the scattering behavior of a polydisperse sample (in this case mixture of monomer and dimer).

Supplement Figure 3

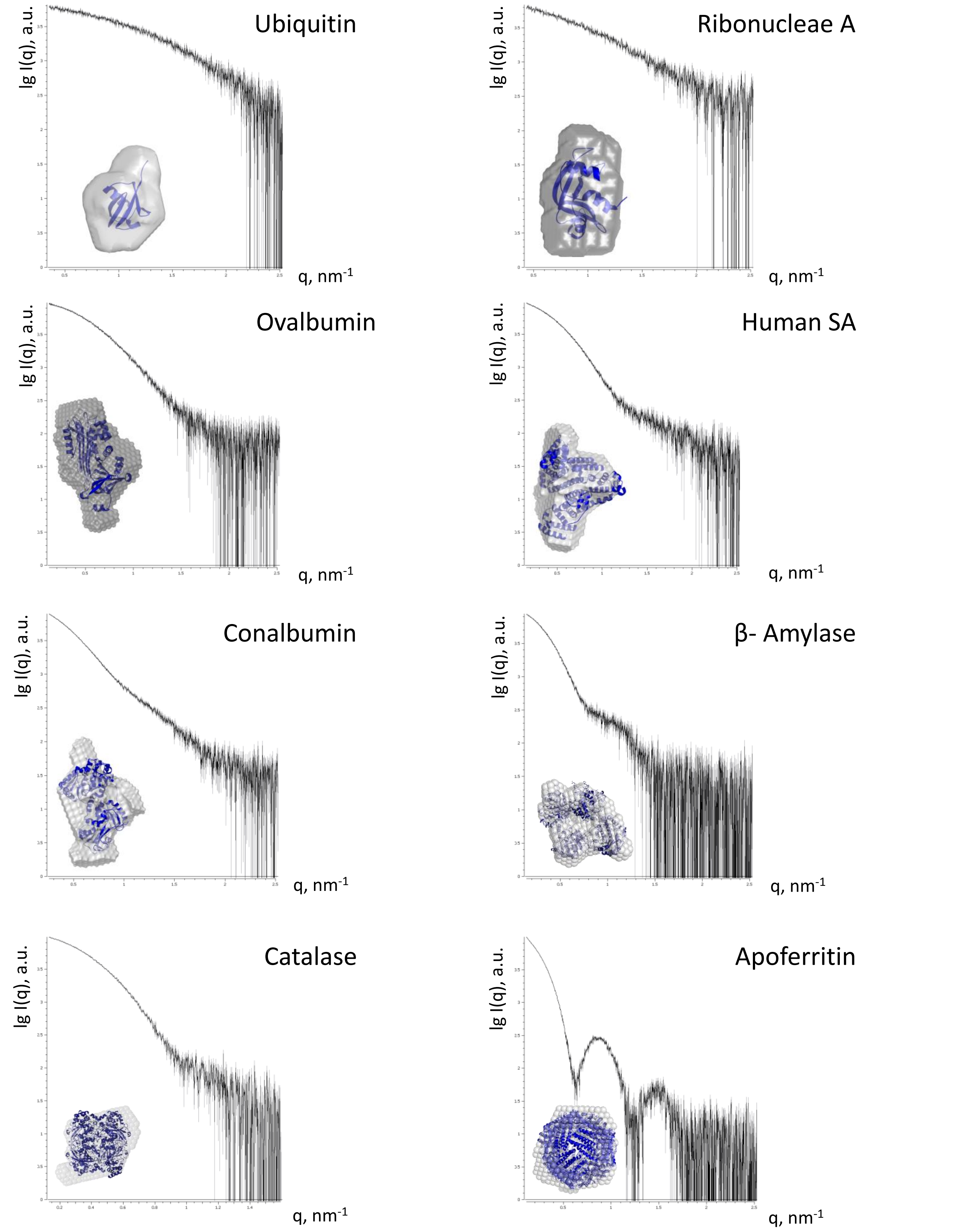

Figure S3 Quality of the SAXS data. Final reduced SAXS profiles were generated by scaling and averaging SAXS data frames corresponding to the SEC elution peak (80-100 frames). This file was further processed and used for the generation of 3D reconstructions. Dammin models are overlaid with the corresponding X-tal structures (pdb codes: Ubiquitin (2ZCC), Ribonuclease A: 1FS3 (1C0B), Ovalbumin (1OVA), Human Serum Albumin (1AO6), Conalbumin (1RYX), ß-Amylase (1FA2), Catalase (4BLC), Apoferritin (2W0O)).

Supplement Figure 4

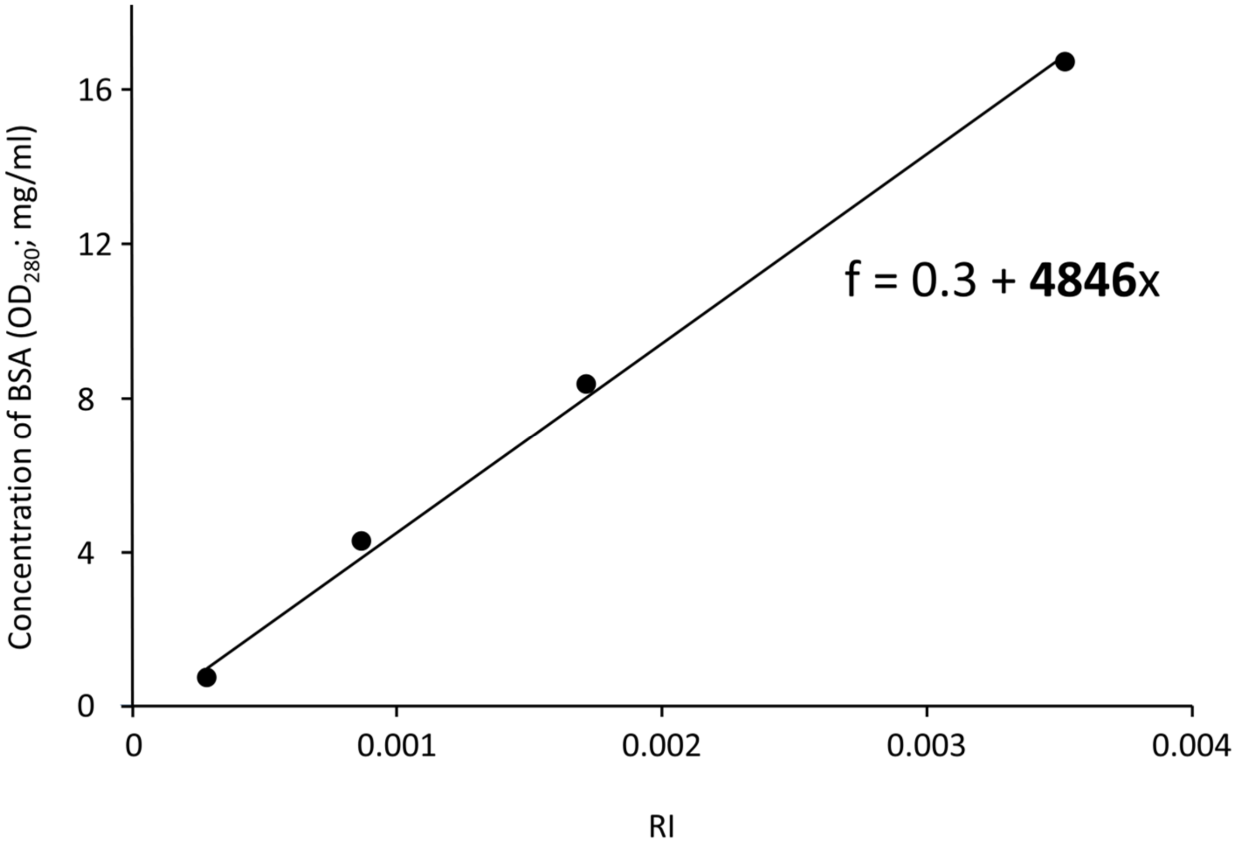

Figure S4 Correlation measurements using RI and OD experiments on the same stock solutions using table-top instruments to derive the correlation constant for proteins.

OD absorption was determined with Thermo Scientific Nanodrops (ND-1000) and correlated with RI measured with a table top RUDOLPH Research Analytical J357 Refractometer for a number of proteins. The correlation constant was derived with linear regression analysis .Future measurements with “tricky samples” will be required to conclude if an assessment of the alteration of this correlation constant leads to valuable information about the sample composition.
